# Facial appearance reveals immunity in African men

**DOI:** 10.1038/s41598-017-08015-9

**Published:** 2017-08-07

**Authors:** Khutso G. Phalane, Catherine Tribe, Helen C. Steel, Moloko C. Cholo, Vinet Coetzee

**Affiliations:** 1 Department of Genetics, University of Pretoria, Pretoria, South Africa; 2 Department of Immunology, University of Pretoria, Pretoria, South Africa

## Abstract

Facial appearance is thought to indicate immunity in humans, but very few studies have tested this relationship directly. The aim of this study was to test the relationship between direct measures of immunity, perceived facial health and attractiveness, and facial cues in African men. We show that men with a stronger cytokine response are considered significantly more attractive and healthy. Men with more masculine, heavier facial features (i.e. muscular appearance) have a significantly higher cytokine response and appear significantly healthier and more attractive, while men with a yellower, lighter, “carotenoid” skin colour, have a marginally higher immune response and are also considered significantly more healthy and attractive. In contrast, more symmetrical, skinnier looking men appeared more attractive and healthier, but did not have a stronger cytokine response. These findings also shed new light on the “androgen-mediated” traits proposed by the immunocompetence handicap hypothesis (ICHH) and we propose that facial muscularity serves as a better estimate of an “androgen-mediated” trait than facial masculinity. Finally, we build on previous evidence to show that men’s facial features do indeed reveal aspects of immunity, even better than more traditional measures of health, such as body mass index (BMI).

## Introduction

Evolutionary theory propose that healthy individuals provide various benefits to their partners, such as reduced risk of infection, increased resources^1^ and ‘good genes’, which are passed on to the offspring^2^. The ‘good genes’ hypothesis states that females choose mates that display traits which indicate high genetic quality, especially in terms of a higher resistance to pathogens^2^. Women are therefore generally expected to choose healthier men^3^, but which traits do they use to assess health and immunity?

Facial attractiveness is highly correlated with perceived health^4, 5^ and is considered to serve as a trait indicating good health and a strong immune response^6, 7^. Several facial cues are known to influence attractiveness and health, namely: symmetry; averageness (how closely the face resembles the majority of other faces in the population); sexual dimorphism (masculinity/femininity); skin colour and texture; and facial adiposity (or facial fatness). Most of the facial cues, apart from masculinity (e.g. manly traits, such as a prominent brow ridge and wider jaws), are consistently associated with attractiveness and perceived health (for review see refs 8–13). Masculinity and facial adiposity are also generally associated with measures of actual health, while support for averageness and symmetry are weak or inconsistent and colour has barely been evaluated^8, 10, 13–15^. Interestingly, recent studies indicate that facial adiposity might be a better indicator of health outcomes than traditional measures of obesity, such as the Body Mass Index (BMI), percentage body fat or waist girth^10, 16^. Here we focus specifically on the relationship between facial appearance and measures of immune response.

Few studies have investigated the link between facial attractiveness and measures of immune response^5–7, 17^. Roberts *et al*.^6^ and Lie *et al*.^7^ found that heterozygosity at the Major Histocompatibility Complex (MHC; also known as Human Leukocyte Antigen [HLA] in humans) positively predict male facial attractiveness in young British and Australian men. The MHC or HLA proteins are cell-surface proteins responsible for the regulation of the immune system. In contrast, studies in African^18^ and Australian^7^ women did not find a significant association between HLA heterozygosity and facial attractiveness. More recent studies found a significant positive relationship between a direct measure of immunity (antibody response after Hepatitis B vaccination) and facial attractiveness in European men^5^, but not women^19^.

Various facial cues might influence the relationship between facial attractiveness and immunity. Roberts *et al*.^6^ found significant positive associations between skin condition, specifically perceived skin health, MHC heterozygosity and facial attractiveness in British men. They did not test which aspect of skin condition might be driving this association, but skin yellowness is a likely candidate. Carotenoids—the yellow, red pigments obtained from fruit and vegetables and deposited in the skin—have previously been shown to increase skin yellowness in African and Caucasian skin^20, 21^. Furthermore, this increase in skin yellowness is considered healthy and attractive in both populations^20, 22, 23^. Carotenoids serve as antioxidants in the body and their levels subsequently reduce after infection^24, 25^, indicating a positive link between carotenoid levels and immunity. Lie *et al*.^7^ found that averageness, but not symmetry or masculinity, mediate the relationship between MHC heterozygosity and facial attractiveness in Australian men, but not women. Roberts *et al*.^6^ also did not find a significant association between measured facial symmetry and MHC heterozygosity. The immunocompetence handicap hypothesis (ICHH) proposes that androgen-mediated traits (e.g. traits influenced by the male sex hormones, such as testosterone) accurately signal condition due to the immunosuppressive effects of androgens^26^. Facial masculinity is generally assumed to serve as such a trait, in that masculine men are predicted to have better immune responses, because only men with strong immune systems are expected to withstand the immunosuppressant effects of the high levels of circulating testosterone necessary to develop masculine features^11^. A recent review, however, concluded that there is little direct evidence of a link between facial masculinity and immunocompetence in humans^11^. Moreover, Rantala *et al*.^5^ found that facial adiposity, but not masculinity, significantly mediates the relationship between immunity and attractiveness in Latvian men.

These studies provide valuable insights into the relationship between facial appearance and immunity, but several gaps still remain. First, the immune system is a complex system, consisting of various different subsystems such as the humoral (or antibody-mediated) response and the cell-mediated response (which mostly involves T cells and responds to any cell that displays aberrant MHC markers)^27^. Antibody-mediated response and HLA heterozygosity (tested in previous studies) represent only a small component of the overall immune response. Cytokines are regulatory proteins that are produced by a wide range of immune cells including macrophages, B and T lymphocytes and mast cells^28, 29^. They play an important role in the interaction between cells of the humoral and cell-mediated immune responses and regulate the body’s response to disease and infection^30^. Eight well studied cytokines were selected for this study, representing the Th1 pathway (“cellular immunity”; e.g. interferon gamma [INF-y], interleukin 2 [IL-2] and Tumour necrosis factor alpha [TNF-α]^31^), the Th2 pathway (“humoral immunity”; e.g. IL-4,-6 and -10^32^), both pathways (e.g. Granulocyte-macrophage colony-stimulating factor [GM-CSF]; review ref. 33) and a chemokine e.g. IL-8^34^). Functional cytokine analysis of Peripheral Blood Mononuclear Cells (PBMCs) after Lipopolysaccharide (LPS) stimulation provides a direct measure of immunocompetence^35^. C-reactive protein (CRP) is an acute phase reactant, commonly used to evaluate infection, tissue injury and inflammation^36^. Functional cytokine analysis and CRP therefore provide a more comprehensive view of immunity. Secondly, previous studies on the topic, focused almost exclusively on white European or Australian populations^5, 10, 37, 38^. To our knowledge, no study has yet tested the association between facial attractiveness and a direct measure of immunity in African men. Thirdly, most previous studies, including some of our own, focused on single facial cues when testing the relationship between facial appearance, health and attractiveness^4, 39, 40^, which disregards the interrelationship between facial cues.

The aim of this study was to test the relationship between two direct measures of immunity (functional cytokine profile and CRP), overall facial appearance (attractiveness and health) and the five main facial cues (adiposity, masculinity, averageness, symmetry, skin colour) in African men.

## Results

According to World Health Organisation standards, 14% of participants were underweight, 69% normal weight, 14% overweight and 3% obese^41^. Facial adiposity was significantly correlated with BMI (r = 0.628, p < 0.0005; indicating that observers were rating facial adiposity appropriately) and showed a curvilinear relationship with perceived attractiveness (F = 4.128, p = 0.019, R^2^ = 0.085; hereafter “attractiveness”) and perceived health (F = 7.137, p = 0.001, R^2^ = 0.138; hereafter “health”). The optimum relative BMI value was 22.4 kg/m^2^ for both attractiveness and health (as calculated from facial adiposity^42^; with under-and-overweight men judged less attractive and healthy). Some of the facial cues were significantly correlated (e.g. adiposity and masculinity; Table 1). Closer inspection revealed non-linear relationships between adiposity and masculinity. The curvilinear relationship between masculinity (x-axis) and adiposity (y-axis) explained more variance (F = 24.038, p < 0.0005, R^2^ = 0.351) than the curvilinear relationship between adiposity (x-axis) and masculinity (y-axis) (F = 18.934, p < 0.0005, R^2^ = 0.301), although both were highly significant. More masculine faces were therefore also considered heavier, but only up to a point, where after further increases in masculinity no longer made the faces appear heavier (Supplementary Figs S1, S2). Squared terms for facial adiposity and masculinity were included in subsequent analysis due to these non-linear relationships.

**Table 1:** Pearson’s correlations between attractiveness and health, facial cues, skin colour, cytokine component and CRP.

	Health	Symmetry	Masculinity	Adiposity	Averageness	CIELab L*	CIELab a*	CIELab b*	Cytokine component	log CRP
Log Attractiveness	0.844***	0.565***	0.407***	0.090	0.400***	0.691***	0.631***	0.699***	0.291^∆^	−0.085
	(92)	(92)	(92)	(92)	(92)	(49)	(49)	(49)	(41)	(68)
Health	1	0.590***	0.519***	0.147	0.406***	0.493***	0.395***	0.490***	0.303^∆^	−0.026
		(92)	(92)	(92)	(92)	(49)	(49)	(49)	(41)	(68)
Symmetry		1	0.463***	−0.034	0.240*	0.234	0.104	0.171	0.208	−0.185
			(92)	(92)	(92)	(49)	(49)	(49)	(41)	(68)
Masculinity			1	0.455***	0.075	0.146	0.108	0.159	0.274	−0.143
				(92)	(92)	(49)	(49)	(49)	(41)	(68)
Adiposity				1	−0.001	0.053	0.003	0.063	0.282	−0.007
					(92)	(49)	(49)	(49)	(41)	(68)
Averageness					1	0.203	0.281^∆^	0.274^∆^	−0.202	00.002
						(49)	(49)	(49)	(41)	(68)
CIELab L*						1	0.849***	0.961***	0.435*	−0.203
							(49)	(49)	(25)	(43)
CIELab a*							1	0.941***	0.363^∆^	−0.108
								(51)	(25)	(43)
CIELab b*								1	0.422*	−0.185
									(25)	(43)
Cytokine component										−0.312
										(25)

^∆^p < 0.1, *p < 0.05, **p < 0.01, ***p < 0.001. N in Bracket.

**Figure 1:**
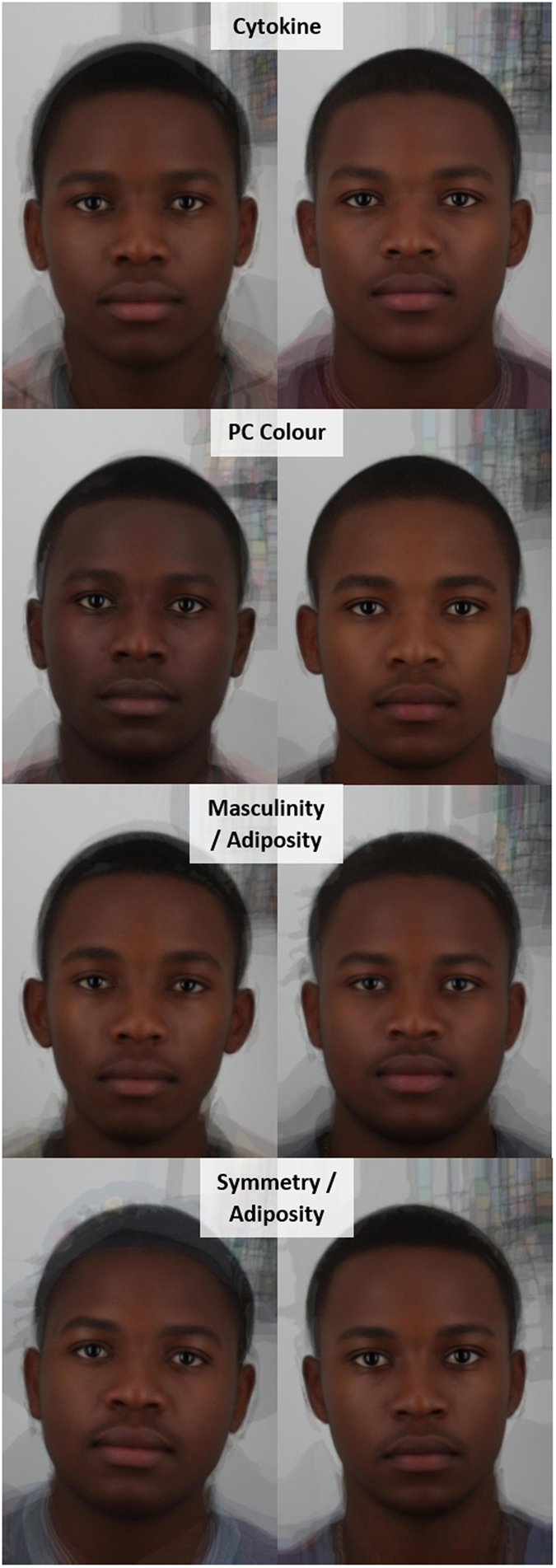
Composite images of cytokine response; PC Colour; the masculinity, adiposity component (PC1); and the symmetry, adiposity component (PC2). Images on the left are composite images of the ten men with the lowest values for that variable, while images on the right are composite images of the ten men with the highest values.

Due to the significant correlations between facial cues, a PCA was conducted on symmetry, averageness, masculinity, masculinity^2^, adiposity and adiposity^2^. Averageness had low communality (0.17) with the rest of the variables and was excluded from the PCA. The PCA produced two principal components with eigenvalue >1, which explained a cumulative variance of 89%. Masculinity (0.90), masculinity^2^ (0.87), adiposity (0.78) and adiposity^2^ (0.75) loaded highly on PC1 explaining 58% of the variance. PC1 is hereafter known as the masculinity, adiposity component, with higher values indicating more masculine, heavier faces (Fig. 1, Supplementary Fig. S3). Symmetry (0.73), adiposity (−0.60) and adiposity^2^ (−0.63) loaded highly on PC2 explaining 30% of the variance. PC2 is hereafter known as the symmetry, adiposity component, with higher values indicating more symmetrical, skinnier faces (Fig. 1, Supplementary Fig. S4). The masculinity, adiposity component was more highly correlated with facial adiposity measures below (r = 0.742, p < 0.0005) than above the median facial adiposity (r = −0.141, p > 0.1), while the symmetry, adiposity component was more highly correlated with facial adiposity measures above (r = −0.669, p < 0.0005) than below the median facial adiposity (r = 0.464, p = 0.001). The masculinity, adiposity component is, therefore, more relevant to underweight and lower normal weight men, while the symmetry, adiposity component is more relevant to upper normal and overweight men.

The average facial colour values (lightness, yellowness and redness) were also highly correlated (Table 1). A PCA of the facial skin colour values produced one principal component with eigenvalue >1, explaining 95% of the variance. Yellowness (0.99), lightness (0.96) and redness (0.96) all loaded highly and positively on PC3, hereafter the colour component. Higher values for this component indicate yellower, lighter and redder skin colour (Fig. 1). There was no significant association between PC3 and PC1 or PC2 (p > 0.1).

Cytokine levels generally increased after LPS stimulation (Supplementary Table S2). The individual unstimulated and LPS stimulated cytokine levels were highly positively correlated with themselves and with each other (Supplementary Table S3), indicating that all cytokine levels showed a similar picture and that individuals with high unstimulated cytokine values generally also had high values after LPS stimulation. A PCA of all the unstimulated and LPS stimulated cytokine levels produced two principal components with eigenvalue >1, which explained a cumulative variance of 85%. All unstimulated and LPS stimulated cytokine values loaded highly and positively on component 1 (>0.73), hereafter known as the cytokine component. Higher values for this component indicate higher unstimulated and LPS stimulated cytokine levels. None of the cytokine values loaded highly on component 2 (<0.51 and >−0.45) with higher loadings for all cytokines on component 1. Component 2 was therefore excluded from further analysis.

**Figure 2:**
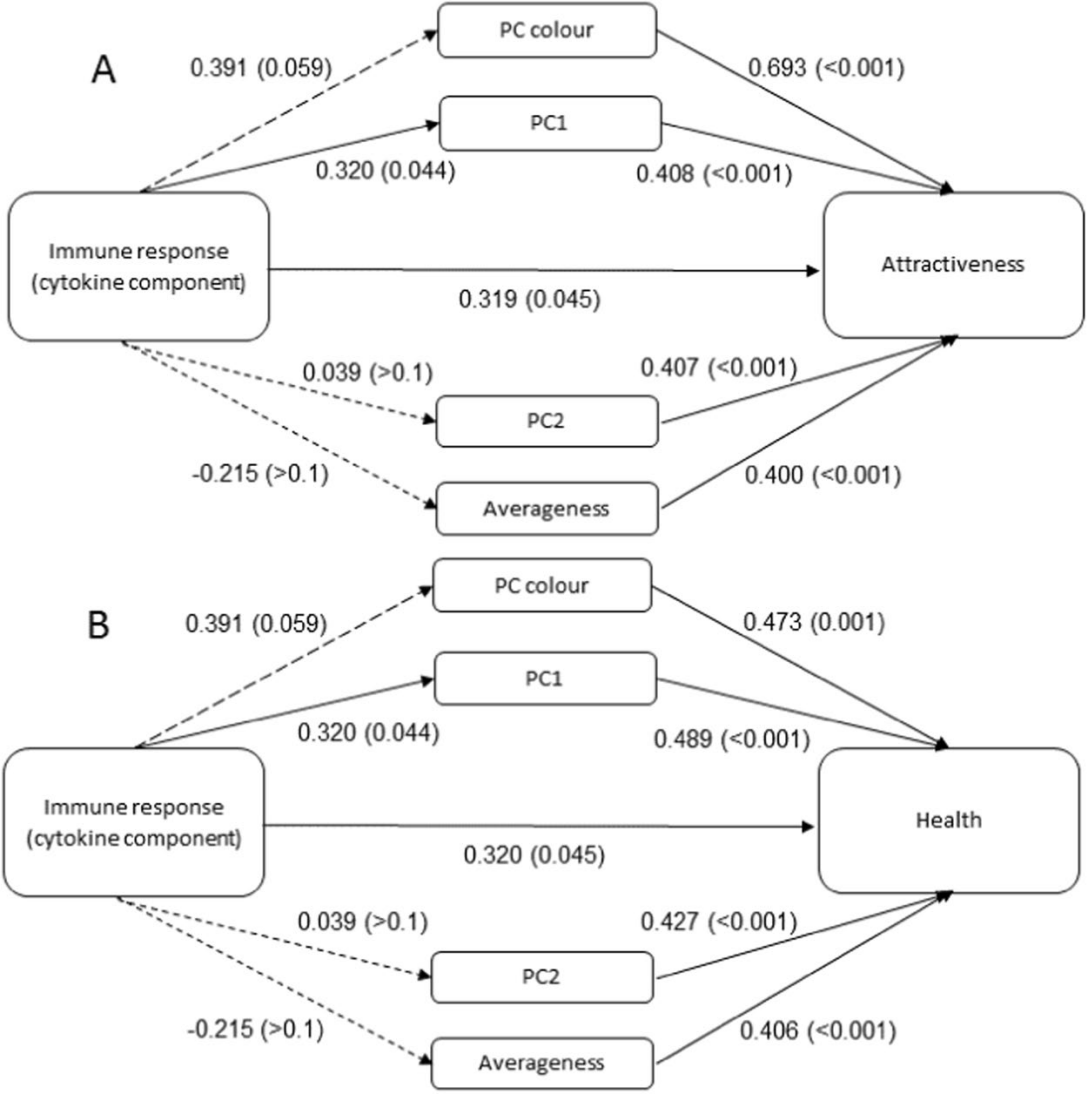
Pearson’s Correlation coefficients for (**A**) log facial attractiveness and (**B**) perceived health. Values indicate Pearson’s correlation coefficients (r) with associated p-values in brackets. Short dashed lines indicate non-significant coefficients (p > 0.05), long dashed lines marginal associations (p < 0.1) and solid lines significant associations (p < 0.05). PC1 = masculinity, adiposity component; PC2 = symmetry, adiposity component.

Age was significantly correlated with the cytokine component (r = 0.343, p = 0.028) and marginally associated with the masculinity, adiposity component (r = 0.197, p = 0.059), but none of the other variables (p > 0.1). We therefore controlled for age in all subsequent correlations involving these two components. The cytokine component was significantly associated with perceived attractiveness and health (Fig. 2), indicating that women consider men with a stronger cytokine response more attractive and healthy. To identify which structural or colour components mediate these relationships we performed separate Pearson’s correlations for all facial and colour components.

The colour component was significantly associated with perceived facial attractiveness and health and marginally associated with the cytokine component (Fig. 2). CIELab b* and CIELab L* were significantly ﻿and﻿ ﻿more strongly﻿ correlated with the cytokine component, attractiveness and health than CIELab a*, which was only significantly associated with facial attractiveness and health (Table 1). Of the three colour variables, skin yellowness and lightness therefore showed the most consistent relationship with both immunity and overall appearance. The correlations between the cytokine component and the spectral values in the palm of the hand closely followed the predicted spectral pattern for carotenoids, especially the carotenoid lycopene, but not the predicted spectral pattern for other human pigments (Supplementary Fig. S5). A similar pattern was not observed in the face, where the correlations between the cytokine component and the spectral values followed the predicted spectral pattern for melanin, indicating the masking effect of melanin in highly melanised skin (Supplementary Fig. S6). The masking effect of melanin on the carotenoid-immune response relationship is further evidenced by a significant association between CIELab b* and the cytokine component above the median for CIELab L* (lighter faces; r = 0.693, p = 0.026), but not below the median for CIELab b* (darker faces; r = −0.245, p > 0.1).

The masculinity, adiposity component (PC1) was significantly associated with the cytokine component, facial attractiveness and health (Fig. 2). In fact, this component was more strongly associated with the cytokine component than BMI was (r = 0.097, p > 0.1). The symmetry, adiposity component (PC2) was significantly associated with facial attractiveness and health, but not with the cytokine component (Fig. 2), even after the removal of one influential outlier (r = 0.189, p > 0.1) Facial averageness was also significantly associated with attractiveness and health, but not the cytokine component (Fig. 2).

Attractiveness and health were highly correlated (Table 1), so similar results were observed for both (Fig. 2), except that the colour component showed a significantly stronger association with attractiveness than with health (Steiger’s Z = 3.48, p < 0.01; Fig. 2). Log CRP was not significantly associated with any of the other variables (p > 0.1), although the relationships were consistently negative.

## Discussion

The aim of this study was to test the relationship between two direct measures of immunity (functional cytokine profile and CRP), overall facial appearance (attractiveness and health) and the five main facial cues (adiposity, masculinity, averageness, symmetry, skin colour) in African men.

We show a significant positive correlation between immune response (e.g. cytokine response before and after immune stimulation), attractiveness and health, indicating that African men with a stronger immune response were judged more attractive and healthy than men with a weaker immune response. This finding is consistent with previous work, which found a significant positive association between Latvian men’s antibody response after Hepatitis B vaccination and their facial and bodily attractiveness^5, 17^. Women with a higher antibody response after Hepatitis B vaccination were not considered more attractive^19^. Previous studies also found a significant positive association between MHC heterozygosity (an indirect measure of improved immunity) and facial attractiveness in young British and Australian men^6, 7^, but not women^7, 18^. Taken together, these findings demonstrate that men (but not women) with a stronger immune response are considered more attractive and healthy than men with a weaker immune response, irrespective of ethnicity.

Previous studies, including some of our own, commonly studied the five main facial cues in isolation. Our results show that some of these facial cues are significantly associated. For example, masculinity and adiposity showed an intricate non-linear relationship, in that men who appeared more masculine also appeared heavier, but only up to a point, where after there was no additional increase in facial adiposity with increased masculinity. Symmetry was also positively associated with both masculinity and averageness, while averageness was somewhat associated with skin redness and yellowness (Table 1). Future studies could test whether these relationships hold in other populations. Studies that test these cues in isolation therefore run the risk of missing the bigger picture or attributing a relationship to one cue, while the relationship might also be explained (and even driven) by another correlated facial cue. A better understanding of these curvilinear relationships might also explain why positive relationships are observed in some studies and negative relationships in others.

Skin colour is strongly and positively associated with overall facial appearance (e.g. attractiveness and health) and marginally associated with immune response. The effect size for the skin colour-immune response relationship (r = 0.391) was larger than the effect size for the significant masculinity, adiposity-immune response relationship (r = 0.320), indicating that the marginal significance was likely due to the small sample size for skin colour values. These findings are consistent with previous work, which found significant positive associations between perceived skin health, MHC heterozygosity and facial attractiveness in British men^6^. The three skin colour components, CIELab b* (yellowness), CIELab a* (redness) and CIELab L* (lightness), were highly correlated as in previous studies in African skin^22, 23^. Nevertheless, we showed that the cytokine component was more strongly associated with skin yellowness and lightness than redness. The correlation between the cytokine component and spectral values of the skin was consistent with the expected pattern for carotenoids in the palm of the hand, especially the carotenoid lycopene. The carotenoid colouration was somewhat masked in the face, due to the presence of melanin, but significant associations between skin yellowness and immune response were observed in lighter faces. These results show that African men with stronger immune responses have yellower skin colours—consistent with what would be expected from a higher carotenoid content—compared to men with weaker immunocompetence. This relationship is, however, somewhat masked in men with darker skin colours.

Consistent with previous studies^20, 23^, both yellowness and lightness played an important role in women’s judgements of male facial attractiveness and health. This preference for a yellower, lighter skin colour can indicate a bias for lighter skin (Colourism)^43^ or a preference for a yellow carotenoid colouration, which is more visible in lighter skin. Two lines of evidence favour the latter. First, we found a somewhat stronger association between attractiveness and skin yellowness than between attractiveness and skin lightness (r∆ = 0.008; although skin lightness was more strongly associated with perceived health [r∆ = 0.003]). This finding is consistent with previous work that also found a stronger association between facial attractiveness and skin yellowness, compared to skin lightness, in African men^23^ and women (22; both r∆ = 0.047). Second, when asked to maximise healthy appearance in a previous study, participants increased skin yellowness more than lightness in both African and Caucasian skin^20^. It is therefore more probable that the preference for a yellower, lighter skin colour is driven by a preference for yellow carotenoid colouration, which is more visible in lighter skin, than for a preference for lighter skin.

The masculinity, adiposity component was significantly and positively associated with cytokine response, attractiveness and health. Heavier, more masculine men therefore have a stronger immune response and are considered more attractive and healthy. At first glance this positive relationship between masculinity, adiposity component and cytokine response seem to contradict the previously observed negative association between antibody response and facial adiposity in Latvian men^5^. There is, however, one important distinction between the two populations: the Latvian cohort contained markedly less underweight men and more overweight-and-obese men (3% and 25% respectively) compared to our African cohort (13% and 17% respectively; Supplementary Fig. S7). If we assume that normal weight men have a stronger immune response than under-and-overweight men (e.g. refs 44 and 45) one would expect a negative relationship in the Latvian cohort and a positive relationship in the African cohort (especially in the lower weight men within this cohort) which is what we found. Moreover, we showed that the masculinity, adiposity component is more relevant to underweight and lower weight men where an increase in adiposity is related to an increase in masculinity. This lack of underweight men in the Latvian sample likely also explains why Rantala *et al*.^5^ observed a negative association between facial adiposity and attractiveness, while this and previous work observed a preference for intermediate weight in men (curvilinear relationship)^10, 13, 42^. Men with a relative BMI of 22.5 kg/m^2^ were considered optimally attractive and healthy, which is consistent with the optimal relative BMI preference of 23.6–24 kg/m^2^ in Scottish men^42^. The masculinity, adiposity component dealt specifically with the left side of this curvilinear relationship, the positive association between facial adiposity and attractiveness in lower weight men. The positive masculinity-immune response component of the relationship is consistent with the previously reported significant positive association between masculinity and antibody response^5^ and to some extend the weak positive association between masculinity and MHC heterozygosity (r = 0.12; 7). Given the generally inconsistent relationship between masculinity and attractiveness^9, 11^, it is not surprising that our positive masculinity-attractiveness relationship is consistent with some, but not all, previous studies on the topic.

The masculinity, adiposity component likely indicates facial features associated with muscularity (i.e. percentage muscle) in men, since: (i) facial adiposity and masculinity were positively correlated in under-and-normal weight men, but not in overweight men, which indicates an important role for muscle not fat; and (ii) African men generally have a higher percentage fat-free mass (mainly muscle mass) than other groups (e.g. Caucasian men and women, African women), indicating that our under-and-normal weight men likely had a substantial amount of muscle^46–48^. Furthermore, testosterone levels are known to increase muscularity and body weight^48, 49^ and have been positively associated with facial masculinity^5, 11, 50^. It is therefore likely that both facial masculinity and adiposity (in under-to-normal weight men) are associated with muscularity and testosterone levels. This is consistent with previous work which found a preference for cues to high testosterone levels in countries with a low Human Development index, such as South Africa^51^. The masculinity, adiposity component was, however, more strongly associated with immune response than facial masculinity or adiposity by themselves, indicating that the masculinity, adiposity (i.e. muscularity) component might be a better estimate of the “androgen-mediated” trait proposed by the ICHH. Indeed, the significant relationships observed between this component and cytokine response is consistent with the ICHH’s key assumption that “androgen-mediated” traits are associated with immune response^11^. We propose that there is indeed a link between immune response and an “androgen-mediated” facial trait in humans, but that the facial trait is *muscularity* (percentage muscle or strength)^52^ and not *masculinity* (i.e. the immune response hypothesis of male facial *muscularity*).

The symmetry, adiposity component (and symmetry by itself) was significantly associated with facial attractiveness and health, but not with the cytokine component. Men with more symmetrical, skinnier faces were therefore judged more attractive and healthy, but they did not have a stronger immune response. These results are consistent with the non-significant association between symmetry and MHC heterozygosity in British and Australian men^6, 7^ and the positive association between symmetry and attractiveness in other populations^7, 9^. Whereas the masculinity, adiposity component dealt with the left side of the curvilinear relationship between facial adiposity and attractiveness (positive association in lower weight men), the symmetry, adiposity component deals with the right side of the curvilinear relationship (negative association in higher weight men). Higher weight men in the upper normal-to-overweight range were considered less attractive, as in previous studies^10, 42^. Higher weight men did not, however, have a weaker immune response as expected from Rantala *et al*.^5^ study, possibly because our population did not contain enough overweight and obese men.

Facial averageness was also significantly associated with attractiveness and health, but not with immune response. This finding is in line with previous studies, which also found more average looking men and women to appear more attractive^7, 9^, but is not in line with the strong positive association found between averageness and MHC heterozygosity in Australian men^7^. Averageness might be associated with certain aspects of immunity, but not others, or there might be population differences in the link between averageness and attractiveness. For example, averageness (e.g. distinctiveness) had the lowest inter-rater reliability of all perceptual measures in our study, indicating that our participants were not able to judge averageness as reliably as the other perceptual judgements. More research is needed to elucidate these relationships.

Very similar results were observed for health and attractiveness due to the high correlation between the two variables, with one notable difference. The colour component was more strongly associated with attractiveness than health. This is somewhat surprising given the marginal association between immune response and colour, but our previous work also found that African participants rely quite heavily on colour, especially skin yellowness, when judging attractiveness in African male and female faces^22, 23, 53^. We found no significant associations between CRP and any of the other variables, although the relationships were consistently negative, with some facial cues (such as skin colour and symmetry) showing larger effect sizes than the correlation between BMI and CRP in a large previous study^48^.

Interestingly, all the facial components, apart from the symmetry, adiposity component, showed a much stronger association with cytokine response than BMI did. This finding is consistent with a growing body of evidence indicating a stronger association between facial features and some health outcomes, than more traditional measures of health, such as BMI^10, 16^. Facial features therefore serve as a very useful predictor of health.

In conclusion, this study builds on previous work to firmly establish the link between facial attractiveness and immune response in men. The work also highlights the intricate relationships between different facial cues and the need to follow a more integrated approach when studying the link between health and facial appearance. We show that two aspects of facial appearance are associated with immune response in African men. Men with a stronger immune response have marginally yellower and significantly more muscular appearing faces and women consider these faces to be more attractive and healthy. In other words, not only do women consider these men healthier, but they are actually healthy. Women also judge men with more symmetrical, average looking and skinnier faces more attractive and healthier, but these men don’t actually have a stronger immune response. These findings shed new light on the ICHH and the “androgen mediated” traits associated with immune response in humans.

## Methods

### Ethics statement

This study was approved by the ethics committee at the University of Pretoria (EC141002-083). All methods were performed in accordance with the relevant guidelines and regulations. Each participant provided written informed consent before taking part in the study.

### Participants

Ninety two African men (mean age = 20.4, SD = 3.0; BMI = 21.7, SD = 3.2) were recruited from the University of Pretoria, South Africa. Participants completed a short questionnaire, including questions on age and ethnicity. Full colour frontal and profile facial photographs were taken with a Canon Eos 40D digital camera under standardized conditions. Participants were asked to maintain a neutral expression. The facial photographs were standardised for orientation and size using Psychomorph and other in-house software. Skin colour measurements were obtained from a subset of individuals (49 participants) on four separate points (right cheek, left cheek, forehead and palm of the hand) using a Konica Minolta CM-2300d Spectrophotometer. The predefined skin areas were measured in CIELab colour space: CIELab L* (luminance axis), CIELab a* (green-red axis), and CIELab b* (blue-yellow axis) and spectral reflectance values (360 to 740 nm). Higher values on the three axes indicate lighter, redder and yellower colours respectively. The measurement aperture was held lightly against the skin to minimize pressure-induced bleaching. All participants were asked to clean these skin areas with hypoallergenic wipes at least 20 minutes before spectrophotometry measurements. Each colour measurement was taken twice and averaged. Colour values for the forehead and cheeks were averaged to provide facial colour values for further analysis. Participants, height and weight were measured and their BMI calculated (weight/height^2^).

### Image Ratings

Twenty African females (mean age = 22.5, SD = 2.2) were recruited from the University of Pretoria Hatfield campus. Each female rated all the male facial photographs for attractiveness, health, symmetry, masculinity, distinctiveness and facial adiposity. Distinctiveness ratings were reverse coded to reflect averageness. Each female participant provided informed consent and completed a short questionnaire with basic demographic (e.g. age, ethnicity) information. The male facial images were presented in a randomised order on a computer screen and female participants were asked to rate each image on separate 7-point Likert scales (1 = very unattractive, 7 = very attractive etc.). All Cronbach alpha values were >0.72 (Supplementary Table S1), which indicates high inter-rater consistency and reliability.

### Immunological analyses

Twenty millilitres of blood was drawn by a qualified phlebotomist from a subset of 68 participants, which included the 20 most attractive and 21 least attractive men that agreed to have their blood drawn. These 41 samples were used for cytokine analysis in order to maximise power and reduce cost. Blood samples were collected in 4 ml heparin BD vacutainer® tubes and processed within 2 hours. PBMCs were isolated at the plasma-Ficoll interphase on Histopaque 1077 Sigma-Aldrich) using standard barrier density gradient centrifugation^54^. Cells were enumerated using Reichert-Jung Microstar light microscopy^55^. Viable cells were stimulated with 0.5 µg/ml of lipopolysaccharide (LPS) and incubated at 37 °C in a CO2 incubator (5%) for 16 hours. LPS is a major component of the outer membrane of Gram-negative bacteria^56^, it stimulates host cells and makes them produce various pro-inflammatory cytokines eliciting strong immune responses^57^. Supernatants were harvested after 16 hours and stored at −20 °C until further use. The levels of eight cytokines: IL-10, -6, -2, -8 and -4, GM-CSF, IFN-γ and TNF-α were evaluated in PBMCs using Bio-Plex Pro™ Assay kits (Bio-Rad Laboratories) on the Bio-Plex Suspension Array System (Bio-Rad Laboratories, Hercules, CA, USA). All samples were analysed undiluted. Bio-Plex Manager Software 6.0 was used for bead acquisition and analysis of median fluorescence intensity. Results are reported as concentration (pg/ml). CRP levels were determined in the serum of a subset of individuals’ using CardioPhase® hsCRP (Siemens) reagents on a BN Prospec Nephelometer (Siemens) as described in Richard and Fogoros^58^. Results are reported as mg/ml. Most CRP values were below 1.0 mg/L, while only 15 participants had CRP values between 1.0 mg/L and 7.3 mg/L.

### Analysis

All analyses were performed in SPSS version 24. Prior to analysis, all variables were examined for accuracy of data entry, missing values, outliers, normality of their distributions and pairwise linearity^59^. All values were normally distributed (two-tailed critical z score = ±3.29, p = 0.001, except facial attractiveness (skewness z = 4.46) and CRP (skewness z = 11.15, kurtosis z = 23.05). Log transformation successfully normalised both distributions (log attractiveness skewness z = 2.07, kurtosis z = −0.57; log CRP skewness z = 2.84, kurtosis z = −0.35). We conducted Principal Component Analyses (PCA) to reduce the number of correlated variables into a smaller set of uncorrelated variables called principal components, thereby revealing the underlying structure of the data^60^. Pearson’s correlations (2-tailed) were conducted to determine the linear relationships between the direct measures of immunity, the facial components/cues and perceived health and attractiveness.

### Data availability

The data will be submitted to the journal Scientific Data.

## Electronic supplementary material

Supplementary information
