# Supplementary material for: Facial appearance reveals immunity in African men
Source: Sci Rep. 2017 Aug 7;7:7443. doi: 10.1038/s41598-017-08015-9 (PMC5547115; doi:10.1038/s41598-017-08015-9)
Supplement: Supplementary file 1 — Supplementary information [file 41598_2017_8015_MOESM1_ESM.pdf]

Supplementary material for:

Facial appearance reveals immunity in African men.

Phalane KG, Tribe C, Steel HC, Cholo MC and Coetzee V.

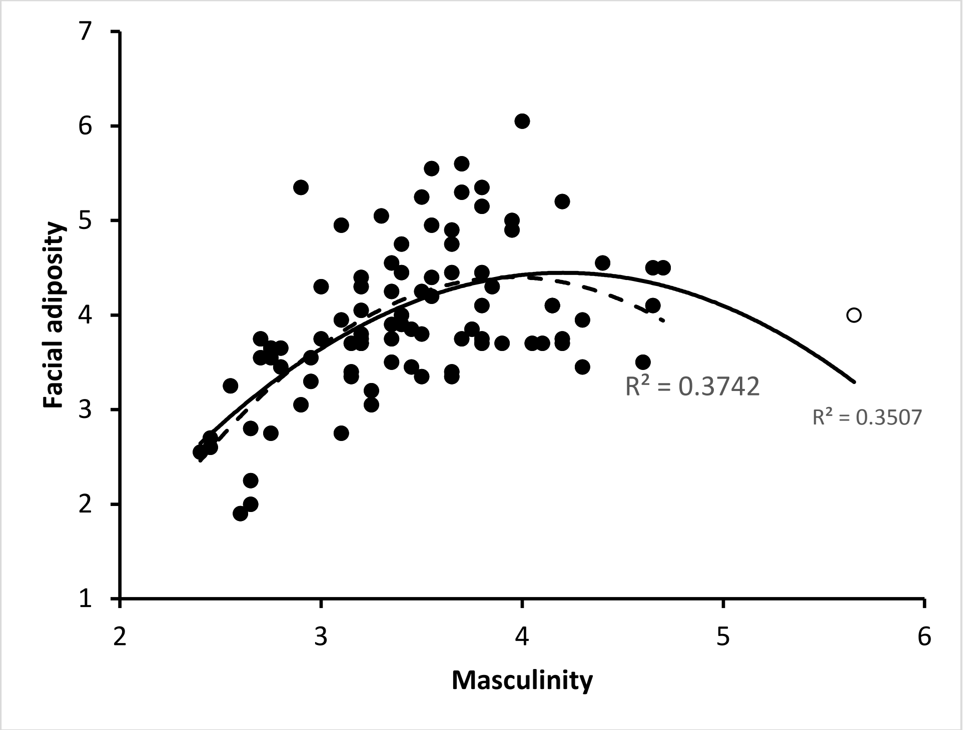

Figure S1: Curvilinear relationship between masculinity and facial adiposity. The curvilinear relationship between masculinity (x-axis) and adiposity (y-axis) explained more variance (F=24.038, p<0.0005, R^2=0.351; dotted line) than the linear relationship between masculinity (x-axis) and adiposity (y-axis) (F=23.457, p<0.0005, R^2=0.207) and the curvilinear relationship between adiposity (x-axis) and masculinity (y-axis) (F=18.934, p<0.0005, R^2=0.301). The removal of one potential outlier (white dot with black outline) only strengthened the relationship (F=26.308, p<0.0005, R^2=0.374; dotted line).

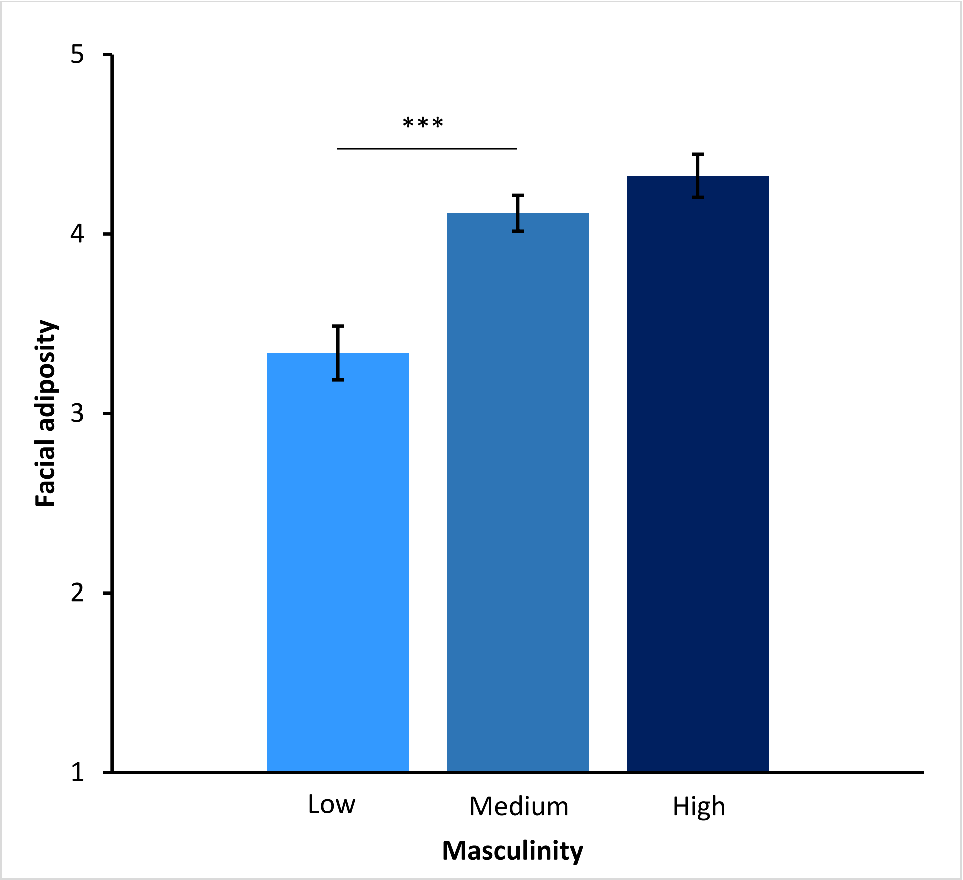

Figure S2. Facial adiposity levels for different masculinity groups. Facial adiposity levels differed significantly between the low and medium masculinity group, but not between the medium and high masculinity group. *** $p<0.0005$. Error bars indicate standard error of the mean.

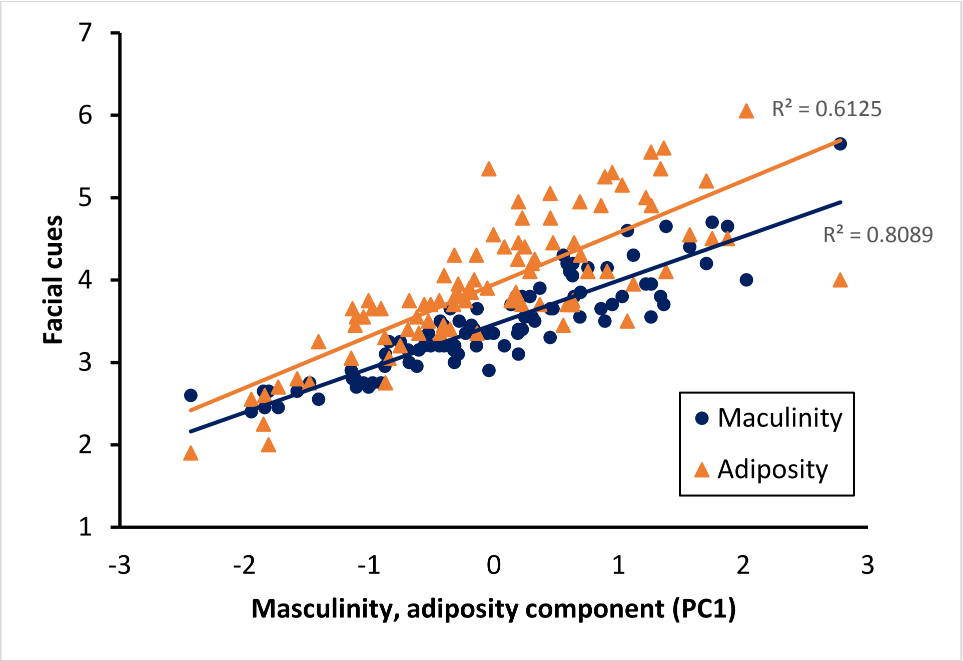

Figure S3. The relationship between the masculinity, adiposity component (PC1), adiposity and masculinity.

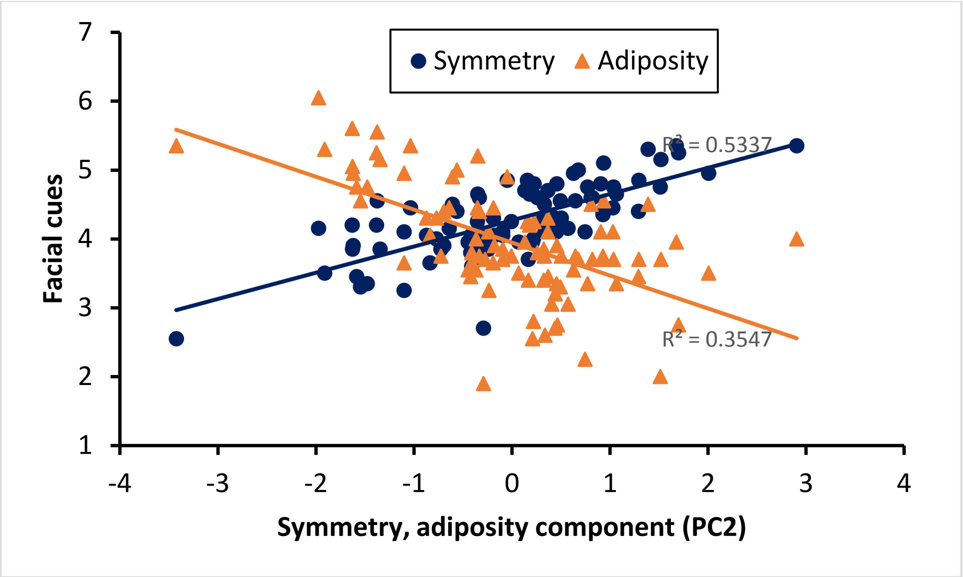

Figure S4. The relationship between the symmetry, adiposity component (PC2) and the facial cues symmetry and adiposity.

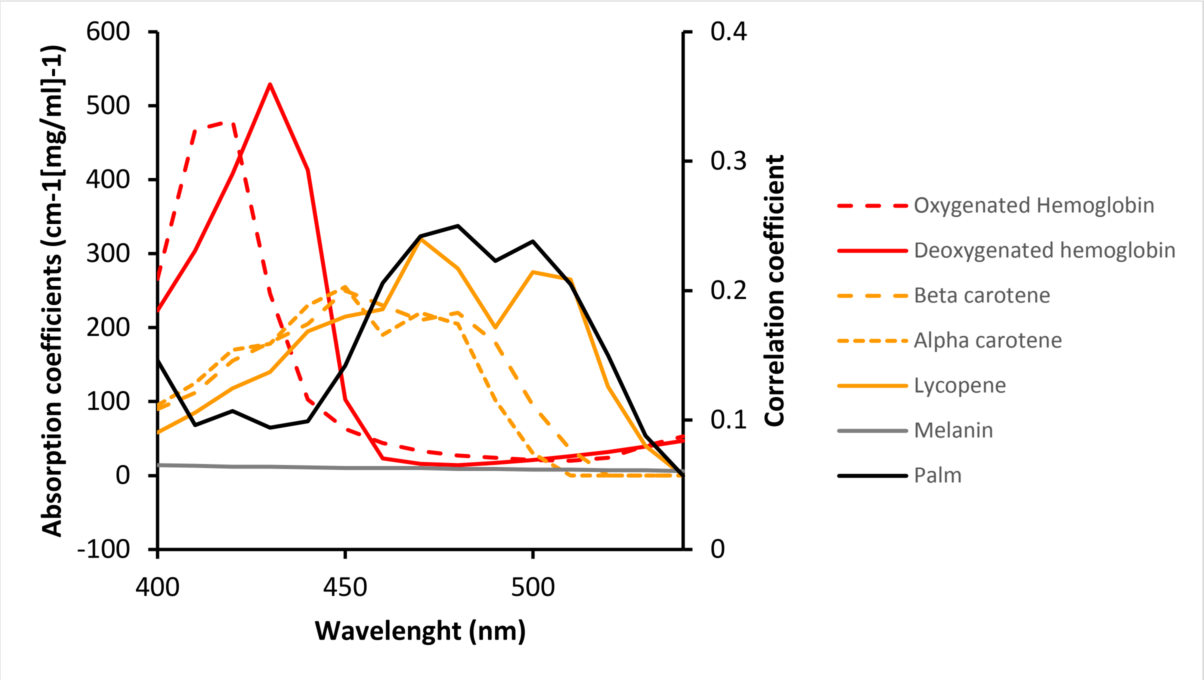

Figure S5: Spearman's correlation coefficients between participant's cytokine component and skin reflectance values in the <u>palm of their hand</u> plotted together with the absorption spectra of common human pigments: Oxygenated hemoglobin, deoxygenated hemoglobin, β-carotene, lycopene and melanin. Correlation coefficient's were reverse scored (by subtracting from 0) to correspond to absorption rather than reflection spectra. Human pigment absorption spectra were obtained from Stephen, Coetzee and Perrett (2011). The correlations between the cytokine component and the spectral values closely followed the predicted spectral pattern for carotenoids, especially the carotenoid lycopene, but not the predicted spectral pattern for other human pigments.

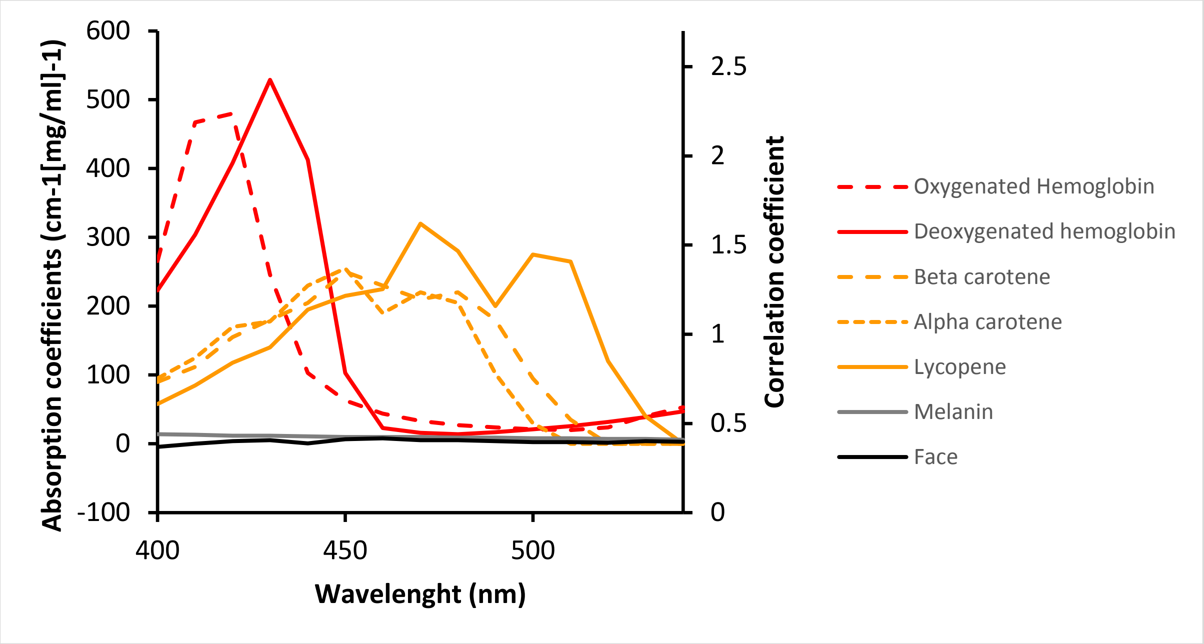

Figure S6. Spearman's correlation coefficients between participant's cytokine component and average facial skin reflectance values plotted together with the absorption spectra of common human pigments. Correlation coefficient's were reverse scored (by subtracting from 0) to correspond to absorption rather than reflection spectra. Human pigment absorption spectra were obtained from Stephen, Coetzee and Perrett (2011). The correlations between the cytokine component and the spectral values were closely associated with the predicted spectral pattern for melanin.

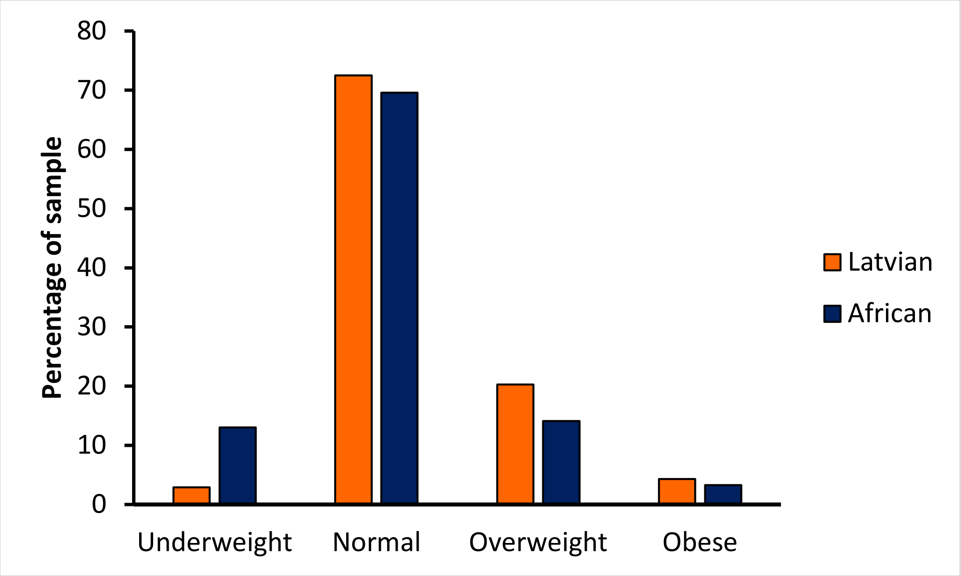

Figure S7. Comparison of the Body Mass Index (BMI) distribution in the current sample (African) and Rantala et al. 2013 (Latvian). BMI classes were calculated according to the World Health Organization criteria: for underweight (BMI <18.5), normal weight (18.5≤BMI<25), overweight (25≤BMI<30), obese (BMI≥30).

Table S1: Cronbach alpha values for rated facial cues

Facial cue	Cronbach’s alpha
Attractiveness	0.94
Health	0.91
Adiposity	0.94
Masculinity	0.82
Symmetry	0.76
Averageness	0.73

Table S2: Average cytokine levels before and after LPS stimulation.

Cytokine	Unstimulated (basal) cytokine level	Stimulated (LPS) cytokine level	Stimulated - Unstimulated
**IL-2**	112.35	241.73	129.38
**IL-4**	88.52	157.17	68.65
**IL-6**	6 107.57	11 539.11	5 431.54
**IL-8**	15 160.09	18 976.78	3 816.69
**IL-10**	864.33	1 725.50	861.17
**GM-CSF**	383.29	696.83	313.54
**IFN-γ**	1 077.80	1 997.28	919.48
**TNF-α**	2 056.37	4 705.26	2 648.89

Table S3: Correlations between individual unstimulated cytokine levels (above diagonal), individual LPS stimulated cytokine levels (below diagonal) and between unstimulated and LPS stimulated cytokine levels (on the diagonal in bold).

	**IL2**	**IL4**	**IL6**	**IL8**	**IL10**	**GM-CSF**	**IFN-y**	**TNF-α**
**IL2**	**.596*****	.955***	.985***	.816***	.899***	.885***	.937***	.968***
**IL4**	.750***	**.654*****	.949***	.841***	.912***	.913***	.952***	.925***
**IL6**	.910***	.779***	**.552*****	.860***	.925***	.870***	.943***	.933***
**IL8**	.629***	.685***	.660***	**.763*****	.851***	.752***	.828***	.749***
**IL10**	.656***	.829***	.708***	.642***	**.660*****	.886***	.902***	.836***
**GM-CSF**	.772***	.887***	.825***	.674***	.772***	**.566*****	.895***	.879***
**IFN-y**	.776***	.916***	.800***	.633***	.811***	.800***	**.676*****	.915***
**TNF-α**	.909***	.736***	.806***	.559***	.583***	.727***	.724***	**.585*****

****p<0.001. N =41. Interleukin 2 (IL2), 4 (IL4), 6 (IL6), 8 (IL8), 10 (IL10); Granulocyte-macrophage colony-stimulating factor (GM-CSF); interferon gamma [INF-y]; and Tumor necrosis factor alpha [TNF-α].*

References

Stephen, I. D., Coetzee, V., & Perrett, D. I. (2011) Carotenoid and melanin pigment coloration affect perceived human health. Evolution and Human Behavior, 32, 216–22
